# Size Constancy in Bat Biosonar? Perceptual Interaction of Object Aperture and Distance

**DOI:** 10.1371/journal.pone.0061577

**Published:** 2013-04-22

**Authors:** Melina Heinrich, Lutz Wiegrebe

**Affiliations:** 1 Department Biology II, Ludwig-Maximilians University Munich, Bavaria, Germany; 2 Graduate School of Systemic Neurosciences, Ludwig-Maximilians University Munich, Bavaria, Germany; 3 Sensory Ecology Group, Max-Planck Institute for Ornithology, Seewiesen, Germany; University of Salamanca- Institute for Neuroscience of Castille and Leon and Medical School, Spain

## Abstract

Perception and encoding of object size is an important feature of sensory systems. In the visual system object size is encoded by the visual angle (visual aperture) on the retina, but the aperture depends on the distance of the object. As object distance is not unambiguously encoded in the visual system, higher computational mechanisms are needed. This phenomenon is termed “size constancy”. It is assumed to reflect an automatic re-scaling of visual aperture with perceived object distance. Recently, it was found that in echolocating bats, the ‘sonar aperture’, i.e., the range of angles from which sound is reflected from an object back to the bat, is unambiguously perceived and neurally encoded. Moreover, it is well known that object distance is accurately perceived and explicitly encoded in bat sonar. Here, we addressed size constancy in bat biosonar, recruiting virtual-object techniques. Bats of the species *Phyllostomus discolor* learned to discriminate two simple virtual objects that only differed in sonar aperture. Upon successful discrimination, test trials were randomly interspersed using virtual objects that differed in both aperture and distance. It was tested whether the bats spontaneously assigned absolute width information to these objects by combining distance and aperture. The results showed that while the isolated perceptual cues encoding object width, aperture, and distance were all perceptually well resolved by the bats, the animals did not assign absolute width information to the test objects. This lack of sonar size constancy may result from the bats relying on different modalities to extract size information at different distances. Alternatively, it is conceivable that familiarity with a behaviorally relevant, conspicuous object is required for sonar size constancy, as it has been argued for visual size constancy. Based on the current data, it appears that size constancy is not necessarily an essential feature of sonar perception in bats.

## Introduction

The representation of object size and its neural encoding is an important function of sensory systems in general. Realistic size estimation over large distances could benefit survival (e.g. in orientation, navigation, foraging, predator avoidance, or intraspecific competition). How organisms perceive the physical object size of distant objects is a fundamental question and not completely answered yet [1], [2]. In vision, three-dimensional space is two-dimensionally represented on the retina along its height and width dimension. Consequently, the extent of the image on the retina in terms of its visual aperture is explicitly encoded. However, when the distance between the observer and the object changes, the visual aperture changes proportionally. “Size constancy” or “size distance invariance” [3], [4], [5] is assumed to reflect an automatic re-scaling of perceived object size with perceived distance. Object distance, however, is not explicitly encoded in the visual system; a mismatch of physical and perceived object distance can lead to a misinterpretation of physical object size and visual illusions [6].

Based on the evaluation of the echoes of self-produced ultrasonic sounds, bats and dolphins achieve detailed acoustic images of their surroundings [7], [8], [9], [10], [11], [12], [13]. But, in contrast to the retina, the sensory epithelium of the auditory system, the basilar membrane, is not arranged along spatial axes. Instead, frequency is explicitly encoded and spatial information must be computed in the central auditory system. Echolocating bats gather information about the physical properties of objects by comparing the returning echoes to their emitted calls [14], [15], [16]. The physical-object properties are encoded in the acoustic parameters of the returning echoes.

In contrast to the visual system where only the aperture is explicitly encoded, the bat auditory system explicitly encodes both, distance and aperture: distance information is encoded by the delay between call emission and the returning echoes [17], [18], [19]. Echo delay and its neural representation were addressed in several studies [20], [21], [22], [23]. It was also shown that object distance is well represented in chronotopically arranged delay-tuned neurons in the bat auditory cortex [22], [24], [25], [26], [27], [28]. The sonar aperture, as the echo-acoustic equivalent to the visual aperture, is defined as the spread of angles of incidence from which echoes impinge on the bat's ears. It was shown that bats can evaluate the sonar aperture independent of echo intensity [13]. Moreover, the sonar aperture is reliably encoded, independent of echo intensity, in the auditory midbrain and cortex [13].

Due to geometric and atmospheric attenuation, echo intensity changes with object distance [22], [29], [30], [31] However, it is an ambiguous distance cue as it not only changes with distance, but also with object size and other physical properties (shape, orientation, texture).

The current study was designed to formally test the hypothesis that bats may combine object-distance and sonar-aperture information to explicitly encode the physical width of a sound-reflecting object independent of object distance.

## Methods

All experiments were conducted under the principles of laboratory animal care and the regulations of the current version of the German Law on Animal Protection. As the experiments are neither invasive nor stressful, they do not require explicit approval. Approval to keep and breed the bats was issued by the Regierung von Oberbayern. The study is divided into a Constancy experiment and a Distance-discrimination experiment. In the Constancy experiment, bats classified the echoes of virtual objects that differed in both distance and physical width. In the Distance-discrimination experiment, the bats were trained to discriminate changes in the distance of a virtual object, while its sonar aperture remained constant. In both experiments, target strength varied proportionally with the spatio-temporal features of the presented objects, unless otherwise stated. Note that here, we use the term ‘target strength’ not in its original (distance-independent) definition, but as a quantification of the peak amplitude in the impulse response (see below).

### Experimental setup

**Figure 1:**
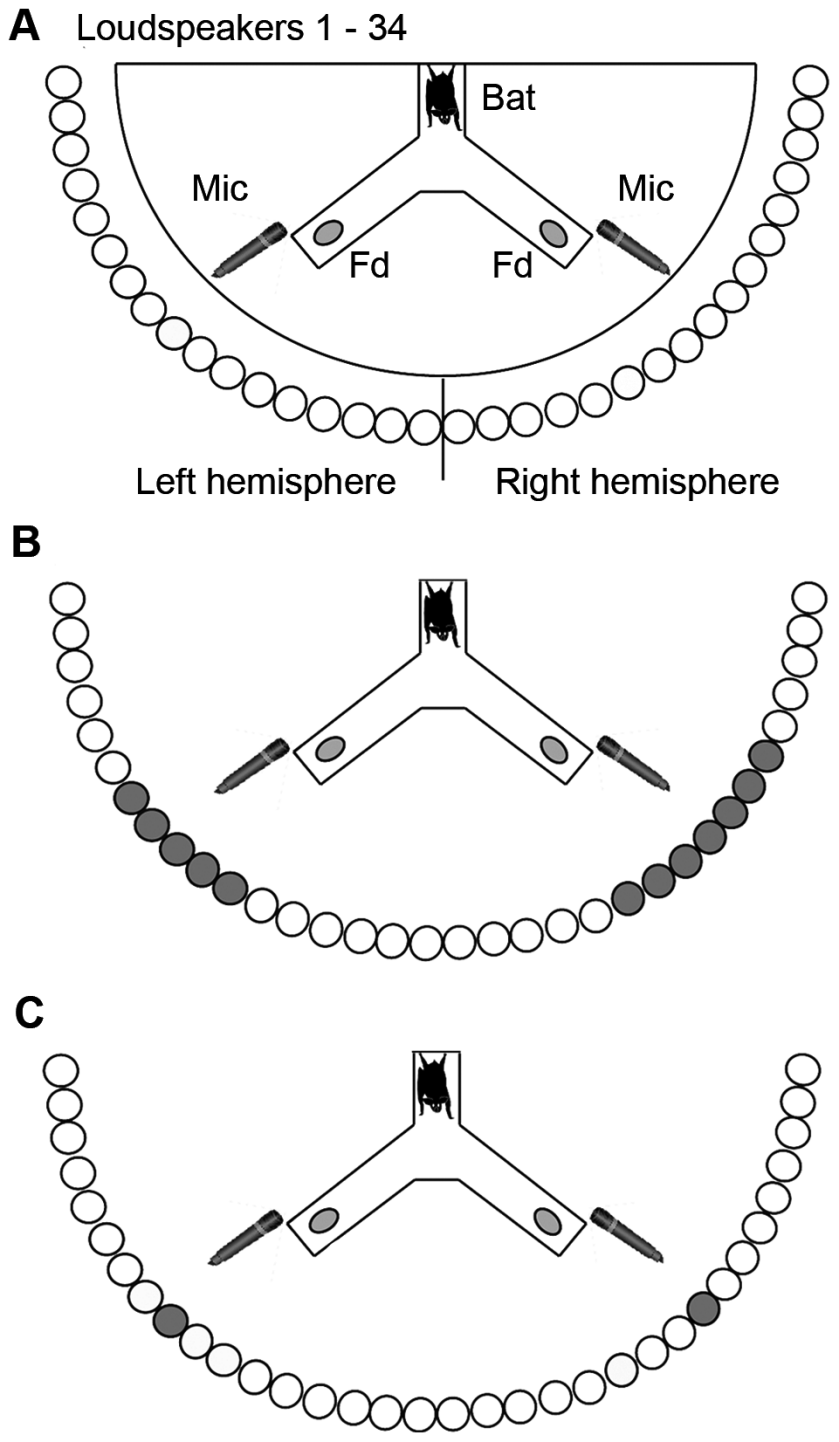
Experimental setup and stimulus presentation. **A:** The experimental setup is depicted from the front, with a bat sitting on the focal point of the y-maze placed in a semicircular wire mesh cage. Indicated are the feeders (Fd), the ultrasonic microphones (Mic), and the 34 ultrasound loudspeakers for virtual-object presentation in the azimuth (open circles). The bar in the center of the loudspeaker array indicates the division into right and left hemisphere. **B:** Presentation of the two different sonar apertures in the Constancy experiment. Left hemisphere: The aperture of the rewarded object (23°) in the trained standard condition is represented by 5 adjacent active speakers (grey). On the right hemisphere the aperture of the corresponding unrewarded object (34°) is represented by 7 adjacent active speakers. **C:** Active speakers of the control experiment on distance discrimination (grey). Here, only one speaker was active in each hemisphere, but echo delay and target strength co-varied.

**Figure 2:**
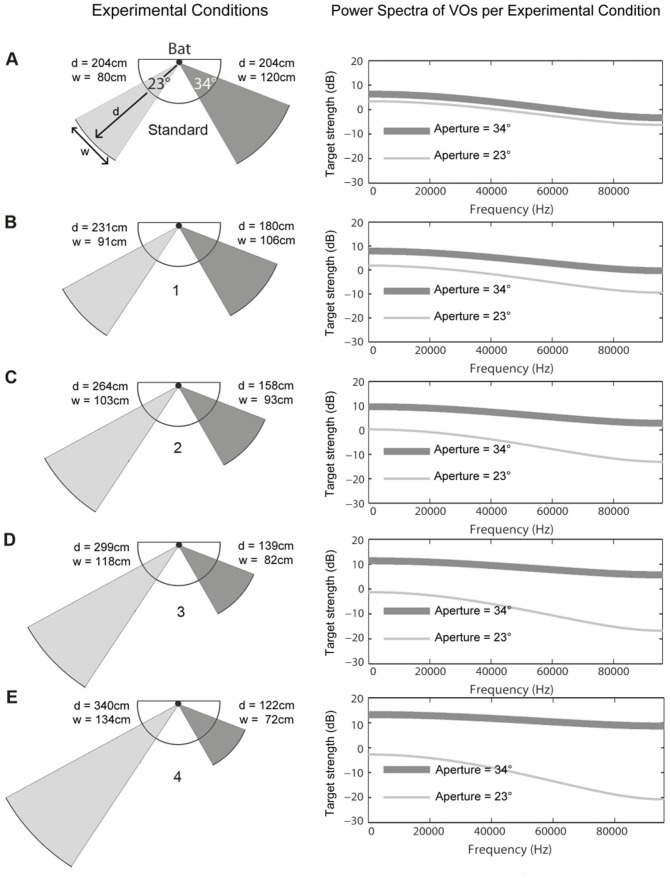
Stimulus presentation in the Constancy experiment. Depicted are the five different experimental conditions of the Constancy experiment (standard condition and test conditions 1–4) in the left column. The power spectra of the virtual objects (VOs) generated by the sum of the active speakers in each hemisphere are plotted on the right. Left column: The bat is indicated as a black dot on the focal point of the maze. The speaker array is indicated by the semicircular line. All VOs depicted in the left hemisphere have an aperture of 23° (light grey); those in the right hemisphere have an aperture of 34° (dark grey). In the standard condition, both VOs are presented at the same distance (d) of 204 cm. The VO with the 23° aperture also provides the smaller object width (w). In the test conditions 1–4, the object with the smaller aperture is presented progressively further away and the object with the larger aperture is presented progressively closer to the bat. As a result, the angular information provided by the aperture does no longer unambiguously code for object width: in the test conditions 2, 3, and 4, the smaller aperture of 23° represents the larger object width. The right column shows the power spectra of the VOs. The solid line shows the change of target strength (dB) with frequency of the VO with the aperture of 34° whereas the dotted line shows that of the VO providing the smaller aperture of 23°. At high frequencies target-strength difference increases even more due to the geometric and atmospheric attenuations that co-vary with distance.

The experimental setup of the current study was the same as in Heinrich et al. [13]. The experiments were performed in a dark, echo-attenuated chamber. The setup inside the chamber consisted of a Y-shaped maze that was placed in a semicircular wire mesh cage (radius = 55 cm). The cage was mounted on a metal post at an angle of 45°. A starting perch was located at the top end of the Y-maze. Each of the other two legs held a feeding device. Two ultrasonic microphones (CO 100K, Sanken) were mounted above the feeders pointing towards the perch. The cage was plane-parallel arranged towards a semicircular loudspeaker array (radius = 71 cm) that consisted of 34 ultrasonic ribbon loudspeakers (NeoCD1.0, Fountek) that were also covered with plain acoustic foam except for the speaker's membrane (0.8×3.8 cm). The array was subdivided into right and left hemisphere (90°) consisting of 17 speakers, each (Fig. 1A). The angular distance between adjacent speakers was 5.6°. Each speaker was calibrated against a 1/8 inch reference ultrasonic microphone (Type 4138, Brüel & Kjær, protective grid removed) at the bat starting perch, perpendicularly oriented towards the speaker axes. To create a compensatory impulse response (IR), the complex spectrum of an ideal bandpass filter (47th-order, finite impulse response, cutoff frequencies: 15 and 94 kHz) was divided by the complex spectrum of the measured IR of each speaker .The real part of the inverse Fourier transform is the compensatory IR. During the experiments, every echo presented over a speaker was first convolved in real time with this speaker's compensatory IR. The convolution with the compensatory IRs ensured that all speakers provided a linear frequency response (between 15 and 94 kHz) as well as a linear phase at the bats starting perch. During the experiments, the bat echolocation calls were picked up by the microphones, amplified (QuadMic, RME) and digitized (HD192, MOTU; two devices with 12 analog-to-digital (AD) and digital-to-analog (DA) channels each and a 424 PCI board, MOTU) at a sampling rate of 192 kHz. To prevent bats from eventually using passive acoustic cues replayed by the speakers (e.g. rustling noises in the high frequency range caused by movements of the bat on the maze), playback was triggered when the recorded signal exceeded a defined threshold. After determining the required echo level (Fig. 2) the echolocation calls were convolved with the compensatory IR of the respective speaker, DA converted, and amplified (AVR 347, Harman Kardon; four devices with seven channels each) before being sent to the speaker. The mismatch between the number of speakers and the AD/DA channels provided by the hardware listed above resulted from the fact that in the experiments not all 34 channels were needed (Fig. 1B and C).

The input-output (I/O) delay of the system (4.3 ms), together with the physical propagation delay from the bat to the microphones (0.9 ms) and from the speakers to the bat (2 ms), added up to 7.2 ms, corresponding to a minimal virtual object (VO) distance of circa 120 cm. In order to prevent masking effects, VOs were presented well beyond the range of physical echoes (e.g. from the cage or the speakers). The experiments were monitored visually (via an infrared observation camera) and acoustically (via heterodyning the microphone signal with a 45 kHz pure tone, playing out the resulting difference frequency across two additional DA converter channels of the HD192 and a headphone amplifier (Terratec Phase 24) into AKG K240 headphones). Experiments were controlled via a graphical user interface from an experimenter seated outside the chamber. Experimental control, data acquisition, and analysis were implemented in MATLAB 7.5 (MathWorks). For the control of the MOTU system, SoundMexPro software (HörTech) was applied.

### Stimuli

Each microphone recorded the animal's ultrasonic calls emitted towards its corresponding hemisphere. The VOs presented on both hemispheres were implemented as simple equidistantly arranged reflectors that could be manipulated along three different parameters: sonar aperture, distance in terms of echo delay, and, physically correct covarying with the particular spatio-temporal parameter, target strength. Sonar aperture was manipulated by changing the number of adjacent speakers presenting an echo. The number of adjacent speakers was always increased symmetrically around the center speaker of each hemisphere. For realistic simulation of object width every single speaker of a VO provided the same sound level so that target strength co-varied with the sonar aperture. Complex spatial interference patterns that could emerge using distant reflectors are discussed in detail elsewhere [13].

The simulated object distance was changed by manipulating the echo delay of the replayed echolocation calls. When object distance was manipulated, the target strength was changed proportionally by taking the atmospheric and geometric spreading losses into account. For the calculation of the atmospheric attenuation an algorithm by Stilz, 2004 [30] was used. The attenuation covered the frequency range between 10 and 96 kHz and was calculated for a relative humidity of 60% and at a temperature of 25°C. For the frequency independent, geometric attenuation the target strength was reduced by 6 dB for each doubling of distance.

### General Procedure

The experimental animals participated on the main experiment (‘Constancy experiment’) and after successful data acquisition on its control experiment (‘Distance discrimination’). Both experiments followed a two-alternative, forced-choice paradigm (2-AFC) with food reward. Before data acquisition, bats were trained to discriminate a rewarded virtual object (RO) from an unrewarded virtual object (UO). The hemisphere for the presentation of the RO was selected pseudorandomly [32]. A decision was indicated by crawling towards one of the two feeders. The test animals were only rewarded when choosing the correct VO. Data acquisition started when a bat's performance reached ≥80% correct choices on five consecutive training days.

### Constancy experiment

For the Constancy experiment, bats were trained to discriminate between two VOs with an aperture of 23° (RO) and 34° (UO) (Figs. 1B and 2A). Both were presented at the same distance (204 cm).

Once the animals had learned this task, test trials were randomly interspersed with a probability of 25%. In these test trials, one of the four test conditions was presented and the animal's spontaneous classification of the test condition was assessed. Note that test trials were always rewarded, independent of the bat's choice. Thus, the spontaneous classification was assessed and learning was deliberately excluded. The four test conditions are illustrated in Fig. 2B–E. In the test conditions, the difference in sonar aperture remained the same, but the object distance was varied such that when the distance of the VO with the smaller aperture exceeded the distance of the VO with the larger aperture (Fig. 2B and Fig. 2C) the VO with the smaller aperture had the larger physical width. Test condition 4 provided the largest difference in object distance between both VOs: here the VO with the smaller aperture had a width that was almost twice that of the VO with the larger aperture (Fig. 2E).

**Figure 3:**
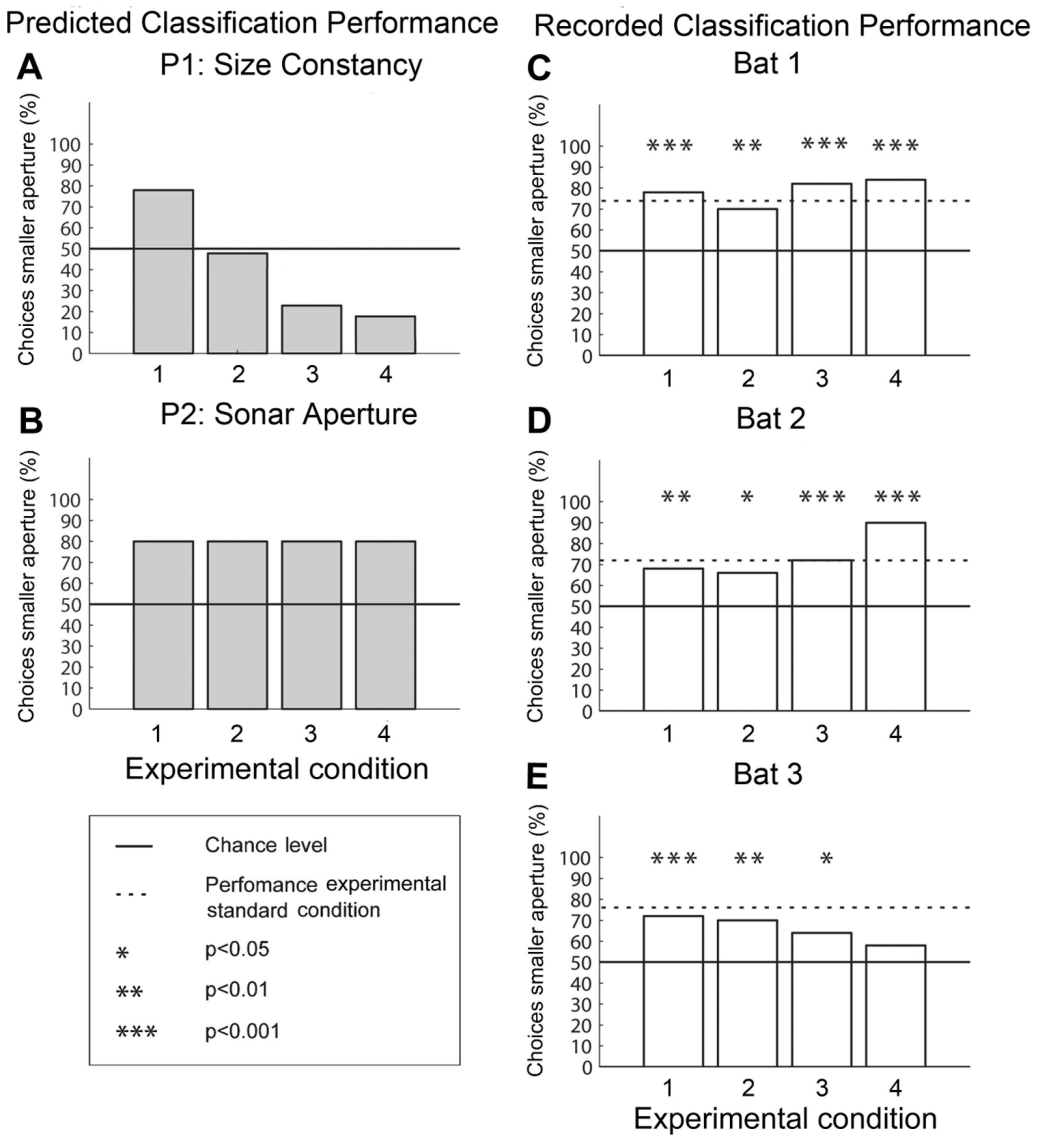
Results of the Constancy experiment. Plotted are the decisions in percent for the virtual object (VO) with the smaller sonar aperture of 23° in the four test conditions. Predictions and recordings of the bat's performance in the Constancy experiment are shown in the left and right column, respectively. If the bats would spontaneously show size constancy with the current paradigm, their classification of the test conditions should look as depicted in **A**. If the bats would spontaneously evaluate the aperture information independent of the accompanying distance information, their classification of the test conditions should look as depicted in **B**. Data in the right column were obtained from three bats. Classification performance is based on 50 trials per test condition. The 50 percent chance level is indicated by the solid line. The dashed line shows the bat's performance in the trained standard condition. The data show that the bats reliably chose the VO providing the smaller aperture independent of the accompanying variation in VO distance.

We predicted that if the bats combined distance- and aperture information to get an estimate of absolute object width, they should classify the VOs with the larger aperture of 34° presented in the test conditions 2–4 as the smaller object (Fig. 3A). On the other hand, if the bats evaluated only the aperture information and did not combine it with the distance information, they should classify the VOs with the smaller aperture of 23° as the smaller VO, independent of object distance (Fig. 3B).

### Data analysis

For analyzing the spontaneous classification performance of the bats in the Constancy experiment, a baseline analysis was applied to verify a reliable classification performance in the standard condition. This was needed because, while all individuals previously reached a stable performance in the standard condition, the performance in the standard condition was not always stable during data-acquisition periods where test conditions were interspersed. To this end, a sliding integration window spanning 30 consecutive trials was applied. The trials of the standard condition lying within this window were analyzed with a binomial cumulative distribution function. The significance threshold was set to p<0.01. When the bat's performance in the standard condition was better than threshold, the test trials within this window were accepted for the further analyses. The analysis window was shifted in one-trial steps. Duplicate test trials were excluded from performance analysis.

The last 50 test trials for each test condition, which met the above criteria, were recruited. Performance was calculated as the decisions for the smaller sonar aperture of 23° in percent. Levels of significance were based on a two-sided binomial distribution (p<0.001, 72%, p<0.01, 68%; p<0.05, 64%).

### Control experiment ‘Distance discrimination’

Echo-acoustic information about object features can only be processed when the acoustic parameters encoding that information are readily perceived. Consequently, the presented acoustic parameters must lie in the perceptual range of the test animals. To rule out the possibility that the bats cannot extract distance information of the presented VOs with sufficient fidelity, a control experiment was performed. To do so, the aperture of the two VOs was equalized and only the bat's sensitivity for differences in VO distance was tested. All three bats that successfully completed data recording in the Constancy experiment participated on the control experiment.

**Figure 4:**
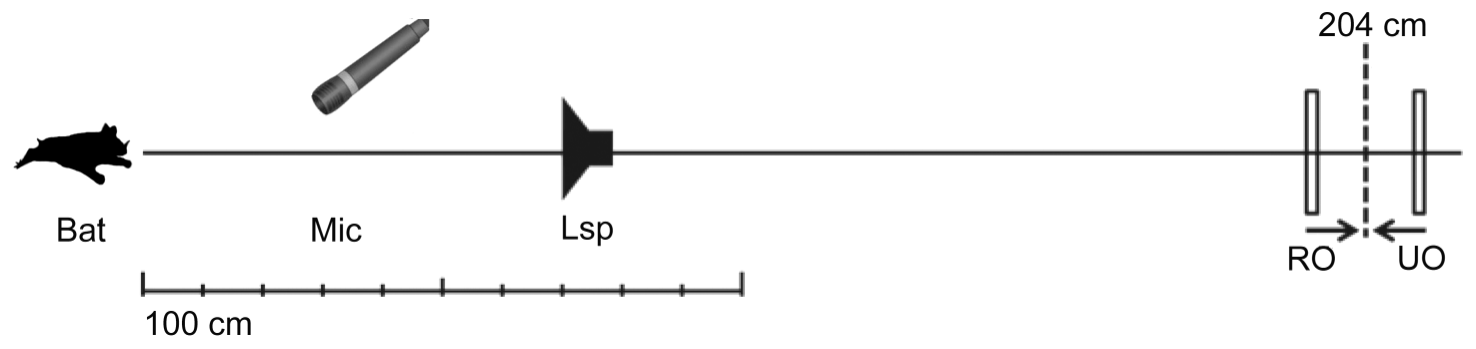
Illustration of real and virtual objects in the Distance-discrimination control experiment. Virtual objects were generated by delaying and attenuating the sounds picked up by the microphone and playing these from the speaker. Thus, the virtual object appears behind the speaker-microphone combination at a distance of 204 cm. The bats were trained to discriminate a distance difference of 17 cm, as indicated by the vertical bars (RO: −8.5 cm; UO: +8.5 cm). Upon successful training, the distance difference was progressively decreased until a threshold could be extracted.

Initially, bats were trained to discriminate a distance difference of 17 cm centered on a reference distance of 204 cm. This corresponds to a difference in echo delay of 1 ms. The RO was always the VO presented at the shorter distance (and thus with the shorter delay). During data acquisition, the distance differences were systematically reduced (Fig. 4). Trials were presented according to a staircase procedure starting with a block of three to five trials with easily discriminable VOs. For each subsequent block, the task difficulty was increased until the bat's performance approached chance level. The distance differences ranged from 17 cm to 1.2 cm (corresponding to echo-delay differences between 1.0 and 0.07 ms).

### Experimental animals

The species used in this study was the Pale Spear-nosed Bat; *Phyllostomus discolor* (Wagner, 1843). This omnivorous phyllostomid bat is found in the rainforests of South-and Central America where it forages on nectar, pollen, fruits, and insects [33]. It emits short (>3 ms), broadband, downward frequency-modulated, multi-harmonic echolocation calls covering the frequency range between 45 and 100 kHz [34], [35]. The individuals came from a breeding colony in the Department of Biology II of the Ludwig-Maximilians-University Munich where they were kept under inverted light/dark conditions. Five adult male *P. discolor* with a bodyweight between 32 and 45 g participated in the experiments. On training days the individuals were kept in a cage (80×60×80 cm). After training sessions the test subjects could fly freely in a room of 12 m^2^ until the next morning. All bats had access to water *ad libitum*. The training was realized in daily sessions that lasted between 15–20 minutes. Five training days were followed by a two day break. The bats were fed with a fruit pulp as reward, consisting of mashed banana, melon, honey, puppy-milk powder, and safflower oil. On days without training, the bats had had access to water, fruit, and mealworms (larvae of *Tenebrio molitor*) *ad libitum*.

## Results

### Constancy experiment

In the Constancy experiment, five bats were successfully trained to discriminate the VO with a sonar aperture of 23° from the VO with an aperture of 34°, both presented at the same distance of 204 cm. Data acquisition, where bats had to classify VOs presented at different distances was successfully completed by three bats. Behavioral results of the Constancy experiment are based on a total of 5762 trials (Bat 1: 1645, Bat 2: 1490, and Bat 3: 2627). The classification performance for the four experimental test conditions is depicted in Figure 3C–E. Here, the decisions for the VO with the smaller aperture in percent are plotted as a function of test conditions 1–4. Each data point is based on the last 50 trials for each test condition (see data analysis section of methods). The solid line indicates chance level for a 2-AFC paradigm, whereas the dashed line indicates the performance level per bat, reached in the standard trials. Except for the classification performance of Bat 3 in test condition 4, the classification performances of the three individuals for all classification tasks were significantly above chance level. When comparing the actual behavioral classification performances of the three test bats to the predicted performances (Fig. 3A and B), it is obvious that none of the three animals showed a switch of the classification performance as would be expected if the animals spontaneously combined aperture- and distance-information to estimate absolute object width.

Instead, the animals' classification is consistent with the hypothesis illustrated in Figure 3B, i.e., the animals evaluated the aperture information of the VOs independent of the distance information.

### Results of the Distance control experiment

All three bats were successfully trained to discriminate a distance difference of 17 cm between two VOs centered at a reference distance of 204 cm.

**Figure 5:**
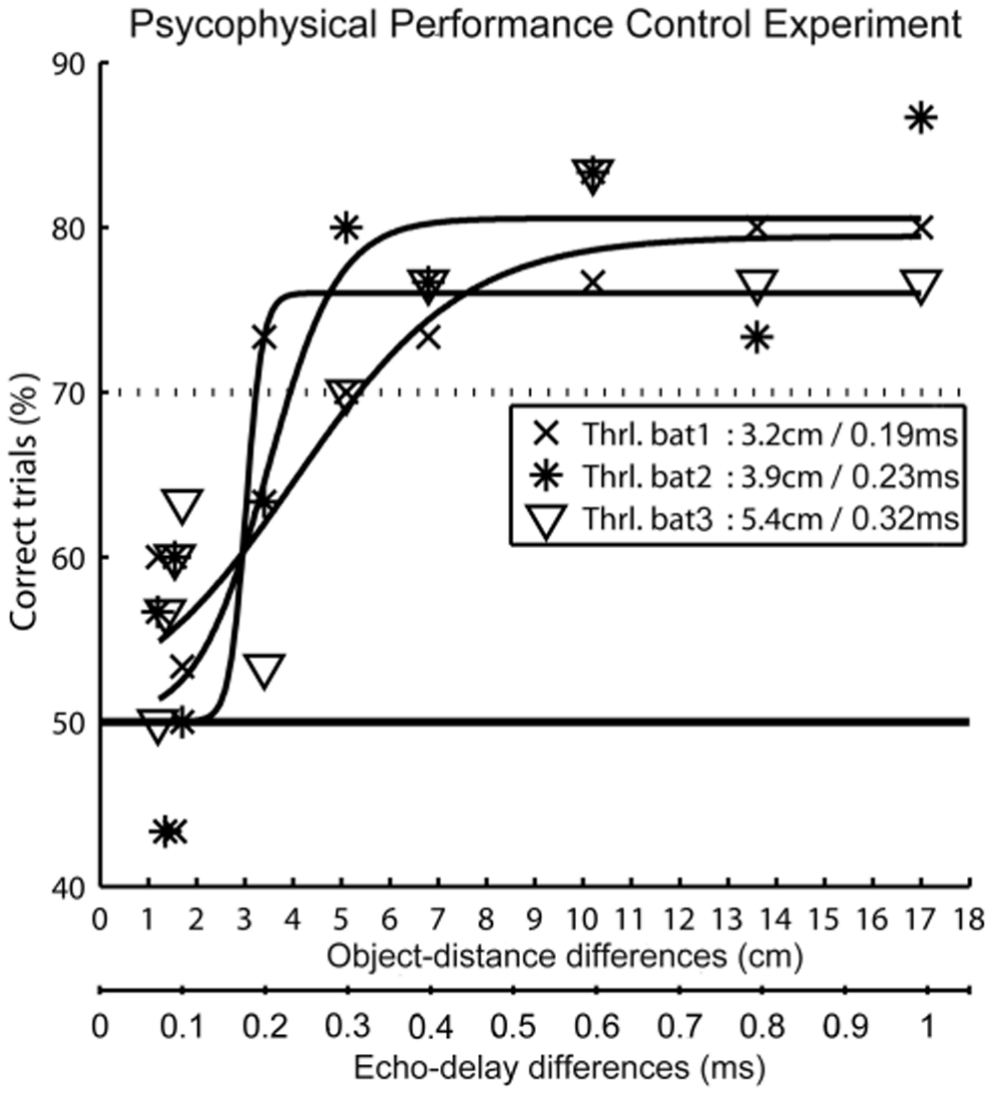
Psychometric functions for distance discrimination. Distance discrimination as a function of distance difference is shown for three bats, marked by different symbols. Data are based on 30 trials per presented distance difference. The upper x-axis shows the distance difference (in cm); the lower x-axis shows the corresponding echo-delay difference (in ms). All bats reliably discriminated distance differences > = 6 cm around a reference distance of 204 cm. Thresholds derived from a sigmoidal fit to the individual psychometric functions and are given in the inset. Chance level (50%) is indicated by the solid line. Level of significance (dashed line) is based on a binomial distribution function (p<0.01).

Individual psychometric functions for the Distance control experiment are plotted in Fig. 5. The upper x-axis shows the presented distance differences (1.2–17 cm) whereas the lower x-axis shows the corresponding echo-delay differences (0.07–1 ms). Performance is plotted as the choices for the VO simulated at shorter distance as percent correct, based on the last 30 trials per presented distance difference. The solid line indicates chance level at 50% for a 2-AFC paradigm. Level of significance was set to 70% (p<0.01) based on a one sided binomial distribution. The psychophysical perception threshold that derived from a sigmoidal fit function was 3.2 cm for Bat 1, 3.9 cm for Bat 2, and 5.4 cm for Bat 3, corresponding to echo-delay differences of 0.188 ms, 0.223 ms, and 0.318 ms for Bats 1–3, respectively. The mean performance of the three bats was 4.16 cm (∼0.247 ms).

The results show that in an active-acoustic virtual-object discrimination task, the sensitivity for differences in object distance of all bats was much better than the object-distance differences as presented in the test conditions 1–4 of the Constancy experiment.

## General Discussion

The bat sonar system theoretically provides the explicit information for the unambiguous encoding of an objects' physical size even when presented at different distances. Therefore, this hypothesis was tested in the Constancy experiment whether echolocating bats of the species *P. discolor* spontaneously assigned absolute width information to VOs by combining distance- and aperture information. To do so, a VO setup was used that allowed for a tight control of all relevant echo parameters, delay, target strength and aperture. The stimulus pairs in the test conditions 1–4 of the Constancy experiment were chosen to create the physically and geometrically correct presentation of VOs allowing the investigation of size constancy.

The results from the Constancy experiment show that the bats significantly chose the VOs providing the smaller sonar aperture of 23° independent of the distance of the VO. This means that no spontaneous assignment of absolute object width could be observed as would be predicted by size constancy (Fig. 3A and C–E).

### Sonar aperture information

For the evaluation of sonar aperture it was already psychophysically and electrophysiological indicated that bats use directional characteristics of their outer ears to evaluate the aperture of ensonified objects [13]. The resulting information can be either monaural spectral cues or binaural-echo disparities [36]. The employment of these parameters for size perception caused by sonar aperture was also supported by the behavioral results of a field study [37].

In Heinrich *et al.* 2011 [13] it was argued that differences in the sonar aperture of two ensonified objects can be monaurally encoded in terms of a difference between the excitation patterns along the tonotopic axis [38]. When variations in echo delay and the accompanying variations in target strength were introduced in the test conditions of the Constancy experiment, this difference between the excitation patterns persisted. For example, if an object with the same aperture at a larger distance was presented, only the delay of the excitation increased and magnitude decreased, but the spectral profile of the excitation pattern would remain unchanged. Thus, if the animals memorized only the excitation-pattern difference, independent of overall excitation and temporal delay, this could explain the animals' classification of the virtual objects in the test conditions.

### Target strength differences

Absolute object width was simulated by combining object distance and aperture. In the current setup, sonar aperture was encoded through the number of speakers coherently radiating the echoes of the bat's emissions. Thus, the target strength of a point receiver at the bat position would increase by 6 dB per doubling of the aperture (i.e., number of radiating speakers). Therefore, the bats could base their discrimination in the standard condition and classifications in the test conditions on target-strength differences, rather than aperture or distance differences.

In the standard condition, both VOs were presented at the same distance to the bats. Consequently the geometric and atmospheric attenuation was identical for both stimuli, and only the aperture differences created target-strength differences. This difference was 3 dB (Fig. 2A, right column) which is lower than perceptual threshold for target-strength discrimination (‘echo intensity’) of 5 dB [13].

The target-strength differences introduced in the test conditions 1–4 were much larger due to the dependence of geometric and atmospheric attenuation on object distance (Fig. 2 B–E). Already in the first test condition (Fig. 2B) the VO with the smaller aperture had also perceivably lower target strength because of the distance difference. But given that the bats based their classifications in the test conditions on the perceptual cues used to discriminate between the VOs in the standard condition, it is unlikely that the bats spontaneously changed its perceptual cue from aperture to target strength. Note that in the test conditions, the bats were rewarded independent of their decision, and, thus, there is no advantage for the bats to switch perceptual cues. Consequently, the bats' classifications are consistent with the hypothesis that they relied exclusively on the aperture cue, and were unaffected by the target strength cues co-varying with the distance cues in the test conditions. We assume this to be true although the target-strength differences were above the perceptual threshold. Interestingly, the human auditory system also relies on perceptual cues other than target strength to evaluate the distance to an external sound source [39]. In that study, it was shown that the direct-to-reverberant ratio of the external sound contributed strongly to its perceived distance. Other potential cues are provided by the (frequency dependent) atmospheric attenuation (Fig. 2) in that more distant sound source or sound reflectors have a stronger low-pass characteristic.

### Distance-discrimination thresholds

The results from the control experiment show on the one hand, that the distance differences as introduced in the Constancy experiment are well above the animals' distance-discrimination thresholds. On the other hand, these thresholds between 3.2 cm and 5.4 cm are slightly higher than those for other bat species (reviews [8], [20]). The distance-discrimination threshold of the closely related bat *Phyllostomus hastatus* [40], for example, was 1.2 cm for real objects presented at a reference distance of either 60 or 120 cm [18], [19]. Data from other bat species also show that thresholds were no larger than 4.1 cm (*Rhinolophus ferrumequinum*, real objects presented at a reference distance of 100 cm) [20], [41].

While most studies have employed real objects for Distance discrimination; the use of VOs in the recent study should not have had negative effects on threshold determination. Using VOs presented at a reference distance of 30 cm, Simmons [19] found distance-discrimination thresholds of 1.0 cm in *Eptesicus fuscus*. These were even slightly better than the 1.2 cm threshold with real objects.

### Simulation of object distance

Compared to other studies using VO setups for distance discrimination, the recent study differed in that we implemented a physically correct distance-dependent attenuation. Besides the echo-delay information, this principally introduced two additional auditory cues the bats could use for solving the task: the target-strength differences of both VOs (the closer VO creates the louder echo) and possible spectral cues. Distance differences and thus delay differences become very small around perceptual thresholds. This is also true for the other acoustic parameters: specifically, at the threshold-distance difference of 4.16 cm, the target-strength difference was only 0.32 dB, which is well below the perceptual threshold of 5 dB [13].

### Multimodal object perception

Doubtlessly, the bat echolocation system is essential for orientation and foraging in complete darkness. Nevertheless, it has limitations and consequently the role of other sensory modalities in bats was also often addressed. *P. discolor* is characterized as mainly nectarivorous or frugivorous, but also feeds on insects or small vertebrates [34]. In addition to echolocation, phyllostomid bat species also rely on olfactory, visual or passive acoustic cues [42], [43], [44]. The fruit-eating bat *Carollia perspicillata* was shown to use primarily olfactory cues for long-range detection and switched to echolocation only to exactly localize fruit items [45]. Other studies have shown that especially frugivorous bats have higher visual acuity [46] as well as better morphological adaptations for vision at low light levels compared to strictly insectivorous bat species [42]. *P. discolor* has relatively large, well developed eyes, suggesting an important role of vision. Indeed it was shown that this species employs both vision and echolocation for object localization and obstacle avoidance [35], [47], [48]. In a flight tunnel experiment that was designed to evaluate the importance the two orientation systems during the object approach, it was found that *P. discolor* preferred visual information at distances larger than 40 cm [47]. This lead to the conclusion, that the use of vision could be more important for object perception at far and medium distances in this species than previously thought. When multiple cues were present, it was found that *P. discolor* chose the sensory orientation system that delivered the most conspicuous object features [34]. The natural habitats of bats are very diverse, including complex structures that cause clutter and can also mask objects of interests. Hence, the more conspicuous object information for size perception over large distances could often be delivered by vision.

Taken together, we assume that while the bats in our experiments were able to evaluate the echo-acoustic aperture- and distance parameters presented in the current experiments, the spontaneous combination of these parameters to create size invariance may lack the ecological relevance.

### Independence of size- and distance-perception?

As indicated above, the discussion of how organisms perceive the physical size of distant objects by vision is still the subject of debate. Haber and Levin [1] challenge the visual size-distance invariance hypothesis in general. They argue that distance perception ‘should develop and be available early in life and in tandem with maturation of locomotors abilities’. For the perception of objects, in contrast, ‘familiarity with the object (acquired from the past) should be the most important variable determining the accuracy of “perceiving” how big the object appears to be’. In the training condition of the current experiment, the bats learned to discriminate between two objects, differing in sonar aperture. In the test trials where distance cues were varied together with aperture cues, the bats continued to choose the VO with the smaller aperture, and appeared to ignore the distance variations. Following the arguments of Haber and Levin [1], distance information in bats is used for acoustically guided locomotion (navigation and orientation) as well as the tracking of prey during pursuit [20], [49], [50], and serves basically the same purpose of distance perception assigned to visually guided locomotion in humans. It is conceivable that the VOs presented in the current experiments lack the behavioral significance to activate ‘object-oriented’ perception [12] (e.g. in terms of e.g. familiarity). This may be the reason why the bats did not show spontaneous size constancy.

We conclude that the perception of angular information as provided by the sonar aperture could be an even more persistent sonar cue than previously thought. For solving the behavioral classification task, bats seemed to ignore variations of object distance (and the covarying amplitude cues) although they were perceptually well resolved by the bats. This lack of sonar size constancy may result from the bats relying on different modalities to extract size information at different distances. An alternative explanation follows Haber and Levin [1] in that familiarity with a behaviorally relevant, conspicuous object is required for size constancy.
